# Impact of surgical margin width on long-term outcomes for intrahepatic cholangiocarcinoma: a multicenter study

**DOI:** 10.1186/s12885-021-08560-7

**Published:** 2021-07-20

**Authors:** Hongzhi Liu, Lianku Lin, Ziguo Lin, Yifan Chen, Qizhen Huang, Lei Ding, Jianying Lou, Shuguo Zheng, Xinyu Bi, Jianming Wang, Wei Guo, Fuyu Li, Jian Wang, Yamin Zheng, Jingdong Li, Shi Cheng, Weiping Zhou, Zhangjun Cheng, Yongyi Zeng

**Affiliations:** 1 Department of Hepatobiliary Surgery, Mengchao Hepatobiliary Hospital of Fujian Medical University, Xihong Road 312, Fuzhou, 350025 Fujian People’s Republic of China; 2 College of Biological Science and Engineering, Fuzhou University, Fuzhou, Fujian China; 3 Department of Hepatobiliary Surgery, The Second Hospital Affiliated to Zhejiang University, Zhejiang, Hangzhou China; 4 Department of Hepatobiliary Surgery, The Southwest Hospital Affiliated to the Army Medical University, Chongqing, China; 5 Department of Hepatobiliary Surgery, Cancer Hospital, Chinese Academy of Medical Sciences, Beijing, China; 6 Department of Hepatobiliary Surgery, Tongji Hospital Affiliated to Tongji Medical College, Huazhong University of Science & Technology, Wuhan, Hubei China; 7 Department of Hepatobiliary Surgery, Beijing Friendship Hospital Affiliated to Capital Medical University, Beijing, China; 8 Department of Hepatobiliary Surgery, The West China Hospital of Sichuan University, Chengdu, Sichuan China; 9 Department of Hepatobiliary Surgery, Renji Hospital Affiliated to Shanghai Jiaotong University, Shanghai, China; 10 Department of Hepatobiliary Surgery, Xuanwu Hospital Affiliated to Capital Medical University, Beijing, China; 11 Department of Hepatobiliary Surgery, The Affiliated Hospital of Chuanbei Medical University, Nanchong, Sichuan China; 12 Department of Hepatobiliary Surgery, Tiantan Hospital Affiliated to Capital Medical University, Beijing, China; 13 Department of Hepatobiliary Surgery III, Eastern Hepatobiliary Surgery Hospital, Secondary Military Medical University, Shanghai, China; 14 Department of Hepatobiliary Surgery, Zhongda Hospital Southeast University, Nanjing, Jiangsu China

**Keywords:** Intrahepatic cholangiocarcinoma, Margin width, Overall survival, Disease-free survival

## Abstract

**Background:**

The objective of this study was to investigate the survival outcomes of surgical margin width in intrahepatic cholangiocarcinoma (ICC).

**Methods:**

Between November 2011 and August 2017, patients who underwent hepatectomy for ICC were collected from 13 major hepatopancreatobiliary centers in China. The survival outcomes for patients who underwent wide margin hepatectomy (WMH) were compared with those who underwent narrow margin hepatectomy (NMH) using the 1:1 propensity score matching (PSM).

**Results:**

Among 478 included patients, 195 (40.8%) underwent WMH whereas 283 (59.2%) underwent NMH. PSM yielded 79 matched patients with similar baseline characteristics. Patients underwent WMH had a significant better OS and DFS compared with those underwent NMH (before PSM: median OS 27 vs 17 months, *P* < 0.05; median DFS 15 vs 8 months, *P* = 0.001, after PSM: median OS 41 vs 22 months, *p* < 0.05; median DFS 16 vs 10 months, *p* < 0.05). However, subgroup analysis based on the AJCC staging system, WMH could only improve the survival outcomes in AJCC I ICC patients (Stage I: OS, DFS, *P*<0.05).

**Conclusions:**

Surgeons should strive to achieve a wide surgical margin for patients with AJCC I ICC to optimize the long-term outcome.

**Supplementary Information:**

The online version contains supplementary material available at 10.1186/s12885-021-08560-7.

## Background

Cholangiocarcinoma (CCA) is a heterogeneous group of malignancies, which derived from any part of the biliary epithelium [1, 2]. According to the location within the biliary system, CCA can be classified into intrahepatic, perihilar, and distal CCA [3]. Intrahepatic cholangiocarcinoma (ICC) is the second most common malignant tumor in liver, and its incidence has been increasing continuously in the past decades [4]. Surgical resection is the most effective treatment for patients with ICC. However, long-term outcome after radical resection is still unsatisfactory [5, 6]. It has been reported that the current 5-year survival after resection of ICC is only 20% ~ 35% [7–9]. Lots of factors, including tumor characteristics and resection factors, are associated with long-term survival after resection of ICC [10, 11]. Among them, surgical margin status and width have attracted many attentions of surgeons and researchers.

Surgical margin status has been reported to be associated with overall survival (OS) and achieving R0 resection is the ultimate objective in resection of ICC [12, 13]. However, the impact of surgical margin width on long-term survival remains controversial. Several studies reported that a gradual better long-term survival was observed as surgical margin width increased [14]. In contrast, some scholars concluded that not all patients with ICC could benefit from a wide margin hepatectomy (WMH) [15]. Assessing the prognostic value of surgical margin width is vital for clinical management of ICC. Given this, we conducted this multicenter study to investigate the impact of surgical margin width on long-term outcomes in ICC patients.

## Patients and methods

### Study cohort

Patients who underwent radical hepatic resection for ICC between November 2011 and August 2017 were identified from a multicenter database that included 13 major hepatopancreatobiliary centers in China (Eastern Hepatobiliary Surgery Hospital of Navy Medical University, Second Hospital Affiliated to Zhejiang University School of Medicine, Mengchao Hepatobiliary Hospital of Fujian Medical University, First Hospital Affiliated to Army Medical University, Cancer Hospital Chinese Academy of Medical Sciences and Peking Union Medical College, Tongji Hospital Affiliated to Tongji Medical College of Huazhong University of Science and Technology, Beijing Friendship Hospital Affiliated to Capital Medical University, West China Hospital of Sichuan University, Renji Hospital Affiliated to Shanghai Jiaotong University School of medicine, Xuanwu Hospital Affiliated to Capital Medical University, Affiliated Hospital of North Sichuan Medical College, Beijing Tiantan Hospital Affiliated to Capital Medical University, Zhongda Hospital Southeast University). Diagnosis of all enrolled ICC patients were histopathologically confirmed. R0 resection was defined as macroscopic and microscopic removal of all tumors [16]. Patients who underwent palliative resection and patients with positive surgical margin, mortality within 1 month of surgery, peritoneal seeding, distant metastasis and incomplete information were excluded. This study was approved by the institutional review board of each participating center.

### Data collection

Data, including patient demographics, perioperative variables, tumor-related clinicopathological characteristics, and follow-up data, were collected using a standardized data sheet. The resectability of the tumor was determined according to the performance status, liver function reserve and tumor imaging features of the patients before surgery. Operative information included the type of hepatectomy, receipt of lymph node dissection, margin status, intraoperative blood loss, transfusion. Postoperative pathological variables included tumor number, size, morphology, grade, vascular/perineural/biliary/adjacent organ invasion, lymph node metastasis, satellite nodules, and surgical margin width. Adjuvant therapy was performed after assessing by a multidisciplinary team. Tumor staging was evaluated according to the 8th edition of the AJCC staging system [17].

Patients were divided into two groups according to the surgical margin width: narrow (< 10 mm) and wide (≥10 mm).

### Follow-up

Patients were regularly followed up every 3–6 months after surgery, during which serum carbohydrate antigen 19–9 (CA19–9) and abdominal CT or MRI were routinely performed. The endpoints of this study were OS and DFS. OS was defined as the interval between the date of surgery and the date of death from any cause or the date of the last follow-up. Disease-free survival (DFS) was defined as the interval between the date of surgery and the first recurrence or the last follow-up.

### Statistical analysis

Categorical variables were expressed as number and percentages, and differences were compared by Chi-square test or Fisher’s exact test. OS and DFS were analyzed by the Kaplan-Meier method, and the log-rank test was used for between-group comparisons. The Cox proportional hazards model was used to identify risk factors of OS and DFS, and variables with statistically significant differences in the univariate analysis were included in the multivariate analysis.

Since patients who underwent WMH and narrow margin hepatectomy (NMH) were not randomly distributed, propensity score matching (PSM) was used to minimize selection bias. The caliper was set at 0.01, and an optimal match ratio of 1:1 was used according to the nearest neighbor method. Statistical analyses were performed using R 3.6.1. A two-tailed *P* value less than 0.05 was considered statistically significant.

## Results

### Patient characteristics

**Fig. 1:**
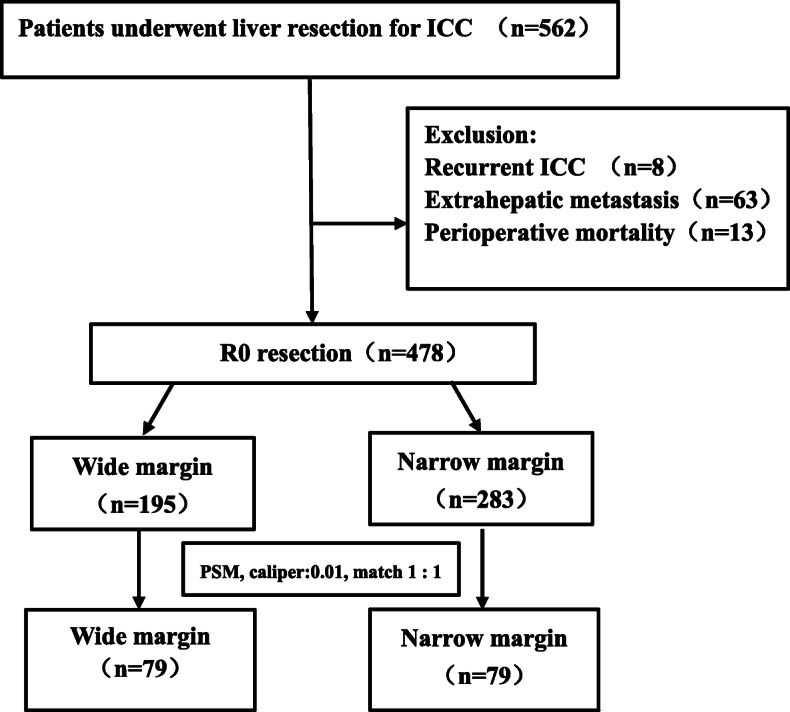
Flowchart of patients’ enrollment

Figure 1 presented the flowchart of patients’ enrollment. The median age of the enrolled 478 patients was 58 years (IQR, 49–64 years) and 287 were male (60.0%). The median tumor size of patients was 6.7 cm, and the majority were single tumor (*n* = 344, 72.0%). In total, 283 (59.2%) underwent NMH, whereas 195 (40.8%) underwent WMH. Several factors, including gender, CA19–9, CEA, blood loss, transfusion, tumor diameter, tumor number, lymph node invasion, gross type, differentiation, satellite, perineural invasion and adjuvant therapy, were associated with margin width (Table 1). Wide margin resection was more frequently performed among patients had a small, single and CA19–9 level raised tumor, and more frequently performed by laparoscopic approach. While age, HBsAg, MVI, and major hepatectomy have no difference between the two groups (*P* > 0.05). Propensity score matching was performed for the above factors that might influence the prognostic analysis. After 1:1 PSM, there were 79 of the 195 WMH patients were matched with 79 of the 283 NMH patients, and all baseline characteristics were compared between the groups.

**Table 1:** Clinicopathological characteristics before and after PSM

	Before PSM			After PSM		
	Wide	Narrow	***P***-Value	Wide	Narrow	*P*-Value
	(*n* = 195)	(*n* = 283)		(*n* = 79)	(*n* = 79)	
**Gender**						
Male	103 (52.8%)	184 (65.0%)	0.010	51 (64.6%)	47 (59.5%)	0.623
Female	92 (47.2%)	99 (35.0%)		28 (35.4%)	32 (40.5%)	
**Age**						
≤ 60 years	115(59.0%)	177(62.5%)	0.489	42(53.2%)	50(63.3%)	0.259
> 60 years	80(41.0%)	106(7.5%)		37(46.8%)	29(36.7%)	
**HBsAg**						
Negative	144 (73.8%)	184 (65.0%)	0.052	55(69.6%)	59(74.7%)	0.594
Positive	51 (26.2%)	99 (35.0%)		24(30.4%)	20(25.3%)	
**CA19–9**						
≤ 200 U/mL	136 (69.7%)	253 (89.4%)	< 0.001	63(79.7%)	65(82.3%)	0.839
> 200 U/mL	59 (30.3%)	30 (10.6%)		16(20.3%)	14(17.7%)	
**CEA**						
≤ 5 μg/L	125 (64.1%)	234 (82.7%)	< 0.001	56(70.9%)	56(70.9%)	1.000
> 5 μg/L	70 (35.9%)	49 (17.3%)		23(29.1%)	23(29.1%)	
**Blood loss**						
≤ 400 mL	141(72.3%)	229(80.9%)	0.036	61(77.2%)	57(72.2%)	0.583
>400 mL	54(27.7%)	54(19.1%)		18(22.8%)	22(27.8%)	
**Transfusion**						
No	148(75.9%)	246(86.9%)	0.003	60(75.9%)	61(77.2%)	1.000
Yes	47(24.1%)	37(13.1%)		19(24.1%)	18(22.8%)	
**Laparoscopic approach**						
No	163 (83.6%)	281 (99.3%)	< 0.001	76 (96.2%)	77 (97.5%)	1.000
Yes	32 (16.4%)	2 (0.7%)		3 (3.8%)	2 (2.5%)	
**Major hepatectomy**						
No	72 (36.9%)	80 (28.3%)	0.058	32(40.5%)	31(39.2%)	1.000
Yes	123 (63.1%)	203(71.7%)		47(59.5%)	48(60.8%)	
**Complications**						
No	137 (70.3%)	233 (82.3%)	0.003	60 (75.9%)	59 (74.7%)	1.000
Yes	58 (29.7%)	50 (17.7%)		19 (24.1%)	20 (25.3%)	
**Tumor size**						
≤ 5 cm	86 (44.1%)	94 (33.2%)	0.020	37(46.8%)	34(43.0%)	0.749
> 5 cm	109 (55.9%)	189 (66.8%)		42(53.2%)	45(57.0%)	
**Tumor number**						
Single	163 (83.6%)	181 (64.0%)	< 0.001	61(77.2%)	60(75.9%)	1.000
Multiple	32 (16.4%)	102 (36.0%)		18(22.8%)	19(24.1%)	
**Lymph node invasion**						
No	140 (71.8%)	250 (88.3%)	< 0.001	67 (84.8%)	69 (87.3%)	0.818
Yes	55 (28.2%)	33 (11.7%)		12 (15.2%)	10 (12.7%)	
**Mass-forming**						
No	38 (19.5%)	116 (41.0%)	< 0.001	28(35.4%)	19(24.1%)	0.164
Yes	157 (80.5%)	167 (59.0%)		51(64.6%)	60(75.9%)	
**Tumor differentiation**						
Well &Moderate	139 (71.3%)	236 (83.4%)	0.002	64(81.0%)	60(75.9%)	0.561
Poor	56 (28.7%)	47 (16.6%)		15(19.0%)	19(24.1%)	
**Satellite**						
No	170 (87.2%)	181 (64.0%)	< 0.001	65(82.3%)	62(78.5%)	0.689
Yes	25 (12.8%)	102 (36.0%)		14(17.7%)	17(21.5%)	
**MVI**						
No	165 (84.6%)	254 (89.8%)	0.124	72(91.1%)	68(86.1%)	0.453
Yes	30 (15.4%)	29 (10.2%)		7(8.9%)	11(13.9%)	
**Perineural invasion**						
No	164 (84.1%)	261 (92.2%)	0.008	66(83.5%)	69(87.3%)	0.652
Yes	31 (15.9%)	22 (7.8%)		13(16.5%)	10(12.7%)	
**p-AT**						
No	141(72.3%)	240(84.8%)	0.001	64(81.0%)	66(83.5%)	0.835
Yes	54(27.7%)	43(15.2%)		15(19.0%)	13(16.5%)	

Abbreviations: *PSM* propensity score matching, *CEA* carcinoembryonic antigen, *CA19–9* carbohydrate antigen 19–9, *HBsAg* hepatitis B surface antigen, *MVI* microvascular invasion, *p-AT* postoperative adjuvant therapy

### Impact of surgical margin width and long-term outcomes

**Fig. 2:**
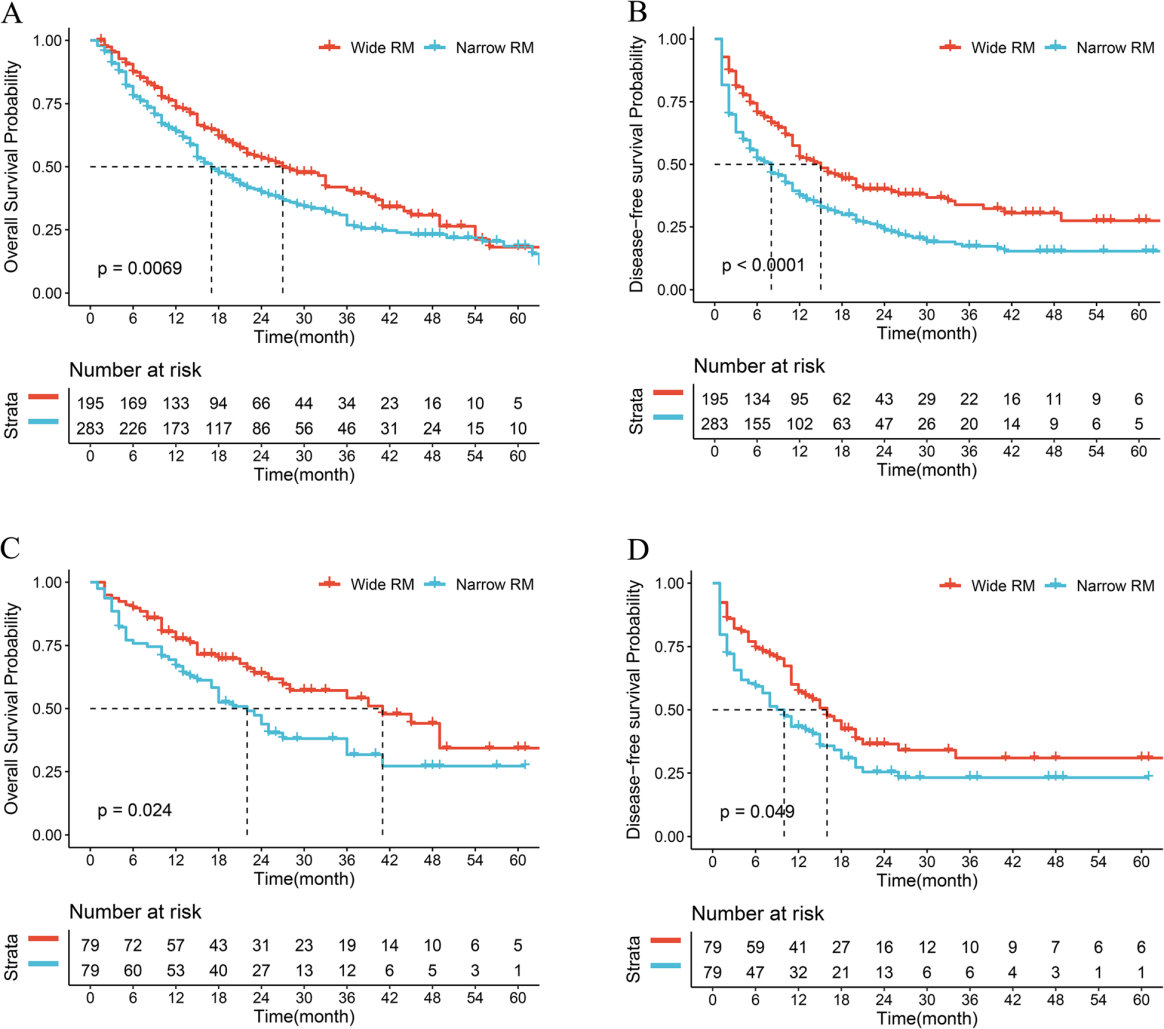
Overall survival (**A**, **C**) and disease-free survival (**B**, **D**) before and after propensity score matching of patients underwent wide margin hepatectomy and narrow margin hepatectomy for intrahepatic cholangiocarcinoma

Among all patients, overall median, 1-, 3-, and 5-year OS was 22 months, 69.20, 36.60, and 26.70%, respectively. Overall median, 1-, 3-, and 5-year DFS was 21 months, 59.80, 41.20, and 37.60%, respectively. Patients underwent WMH had a longer median OS compared with patients undergoing NMH (33 vs 18 months, *P* < 0.05; Fig. 2 A). The 1-, 3-, and 5-year OS in WMH were also higher significantly than in the NMH (76.10%, vs 66.22, 56.10% vs 39.86, 50.24% vs 37.16%, all *p* < 0.05, respectively). Meanwhile, patients underwent WMH had a longer median DFS compared with patients undergoing NMH (16 vs 8 months, *P* < 0.001; Fig. 2 B). The 1-, 3-, and 5-year DFS in WMH were also higher significantly than in the NMH (58.05% vs 40.88, 45.85% vs 28.04, 36.59% vs 27.36%, all *p* < 0.001, respectively).

After 1:1 PSM, the median, 1-, 3-, and 5-year OS of patients in the WMH were still better than that in NMH (40 vs 21 months, 81.51% vs 67.23, 63.87% vs 40.34, 57.14% vs 40.34%, all *p* < 0.05, respectively; Fig. 2 C). Similarly, the median, 1-, 3-, and 5-year DFS of patients in the WMH were also better than that in NMH (17 vs 9 months, 60.50% vs 45.38, 46.22% vs 34.45, 45.38% vs 33.61%, all p < 0.05, respectively; Fig. 2 D).

### Univariate and multivariate cox analyses of OS and DFS in patients with intrahepatic cholangiocarcinoma

Before PSM, univariate analysis identified surgical margin width was associated with OS and DFS (all *P* < 0.05). Additionally, multivariable analysis showed that surgical margin width was an independent prognostic factor affecting OS and DFS (Table S1). After PSM, univariate analysis identified surgical margin width was associated with OS and DFS (all P < 0.05). However, multivariable analysis showed that surgical margin width was an independent prognostic factor affecting OS but not DFS (Table 2).

**Table 2:** Univariate and multivariate analysis of overall survival and disease-free survival for patients with intrahepatic cholangiocarcinoma after PSM

Characteristic	Variables	OS				DFS				
		Univariate analysis		Multivariate analysis		Univariate analysis		Multivariate analysis		
		HR (95%*CI*)	*P* value	HR (95%*CI*)	*P* value	HR (95%*CI*)	*P* value	HR (95%*CI*)	*P* value	
Gender	Female vs Male	0.72(0.45–1.14)	0.158			0.77(0.51–1.16)	0.217			
Age (y)	≤60 vs > 60	0.92(0.59–1.43)	0.706			0.71(0.48–1.06)	0.098			
HBsAg	Negative vs Positive 1.08(0.67–1.75)		0.750			1.08(0.70–1.66)	0.738			
ECOG score	≤2 vs > 2	1.00(0.64–1.55)	0.986			0.80(0.54–1.18)	0.255			
CA19–9 (U/ml)	≤200 vs > 200	1.23(0.71–2.14)	0.458			0.88(0.53–1.47)	0.625			
CEA (ng/ml)	≤5 vs > 5	1.03(0.63–1.67)	0.912			0.91(0.59–1.39)	0.653			
Blood loss (ml)	≤400 vs > 400	1.02(0.61–1.71)	0.947			0.74(0.46–1.19)	0.216			
Transfusion	No vs Yes	1.15(0.69–1.90)	0.600			0.61(0.37–1.02)	0.058			
**Laparoscopic approach**	No vs Yes	1.39(0.34–5.70)	0.644			0.74(0.18–3.00)	0.671			
Major hepatectomy	No vs Yes	1.34(0.84–2.15)	0.215			1.02(0.69–1.52)	0.920			
**Complications**	No vs Yes	1.06(0.65–1.73)	0.809			0.87(0.56–1.37)	0.562			
Resection margin (cm)	≤1 vs > 1	1.65(1.06–2.58)	0.026	1.60(1.03–2.50)	0.039	1.49(1.01–2.20)	0.046			
Tumor size (cm)	≤5 vs > 5	1.33(0.85–2.08)	0.214			1.15(0.77–1.70)	0.497			
Tumor number	Solitary vs Multiple	1.31(0.81–2.12)	0.267			1.14(0.73–1.80)	0.558			
Lymph node invasion	No vs Yes	2.03(1.15–3.57)	0.014	2.24(1.27–3.95)	0.005	1.15(0.65–2.02)	0.638			
Mass-forming	No vs Yes	1.17(0.73–1.87)	0.524			1.33(0.87–2.05)	0.187			
Tumor differentiation	Well &Moderate vs Poor	1.33(0.85–2.08)	0.214			0.80(0.48–1.34)	0.399			
Satellite	No vs Yes	1.27(0.77–2.11)	0.352			1.32(0.84–2.09)	0.231			
MVI	No vs Yes	2.03(1.12–3.69)	0.02			2.42(1.39–4.22)	0.002	2.67(1.50–4.74)	0.001	
Perineural invasion	No vs Yes	0.94(0.50–1.79)	0.860			0.69(0.38–1.26)	0.232			
p-AT	No vs Yes	0.38(0.18–0.83)	0.016	0.39(0.18–0.85)	0.018	0.73(0.43–1.24)	0.244			

Abbreviations: *PSM* propensity score matching, *CEA* carcinoembryonic antigen, *CA19–9* carbohydrate antigen 19–9, *HBsAg* hepatitis B surface antigen, *MVI* microvascular invasion, *p-AT* postoperative adjuvant therapy, *OS* overall survival, *DFS* disease-free survival, *HR* hazard ratio

### Subgroup analysis based on clinicopathologic feature

**Fig. 3:**
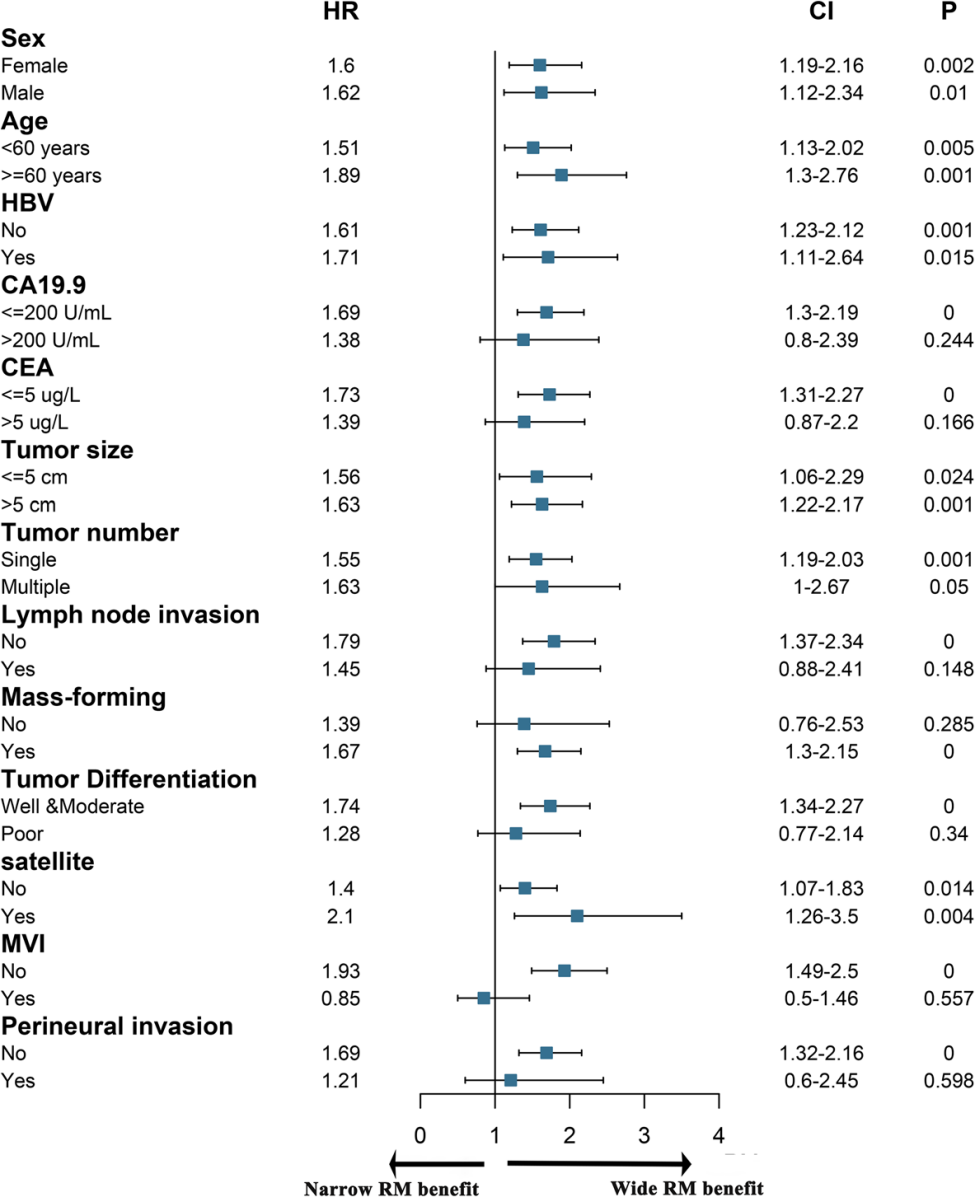
Forest plot of subgroup analysis stratified by risk factors according to disease-free survival

**Fig. 4:**
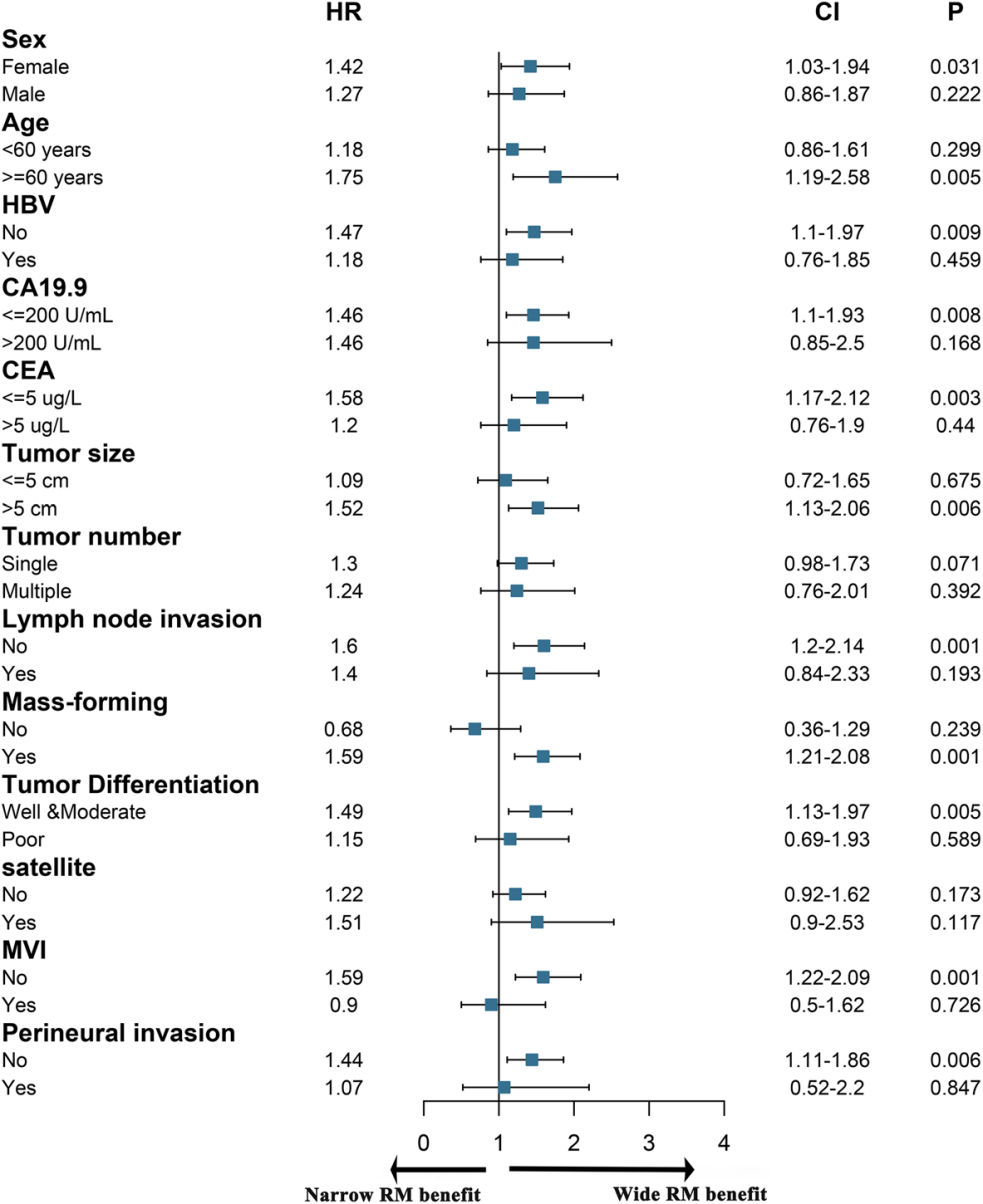
Forest plot of subgroup analysis stratified by risk factors according to overall survival

To identify the optimal ICC patients for WMH, subgroup analysis was conducted based on clinicopathologic feature. As shown in Fig. 3, The following factors may benefit DFS of the patients who underwent WMH: CA199 ≤ 200 U/mL, CEA ≤ 5μg/L, no lymph node metastasis, MF type, mild tumor differentiation, no MVI, and no perineural invasion (Fig. 3). The following factors may benefit OS of the patients who underwent WMH: female, ≥60 years, non-HBV infection, CA199 ≤ 200 U/mL, CEA ≤ 5μg/L, tumor size > 5 cm, no lymph node metastasis, MF type, mild tumor differentiation, no MVI, and no perineural invasion (Fig. 4).

### Subgroup analysis based on AJCC staging system

**Fig. 5:**
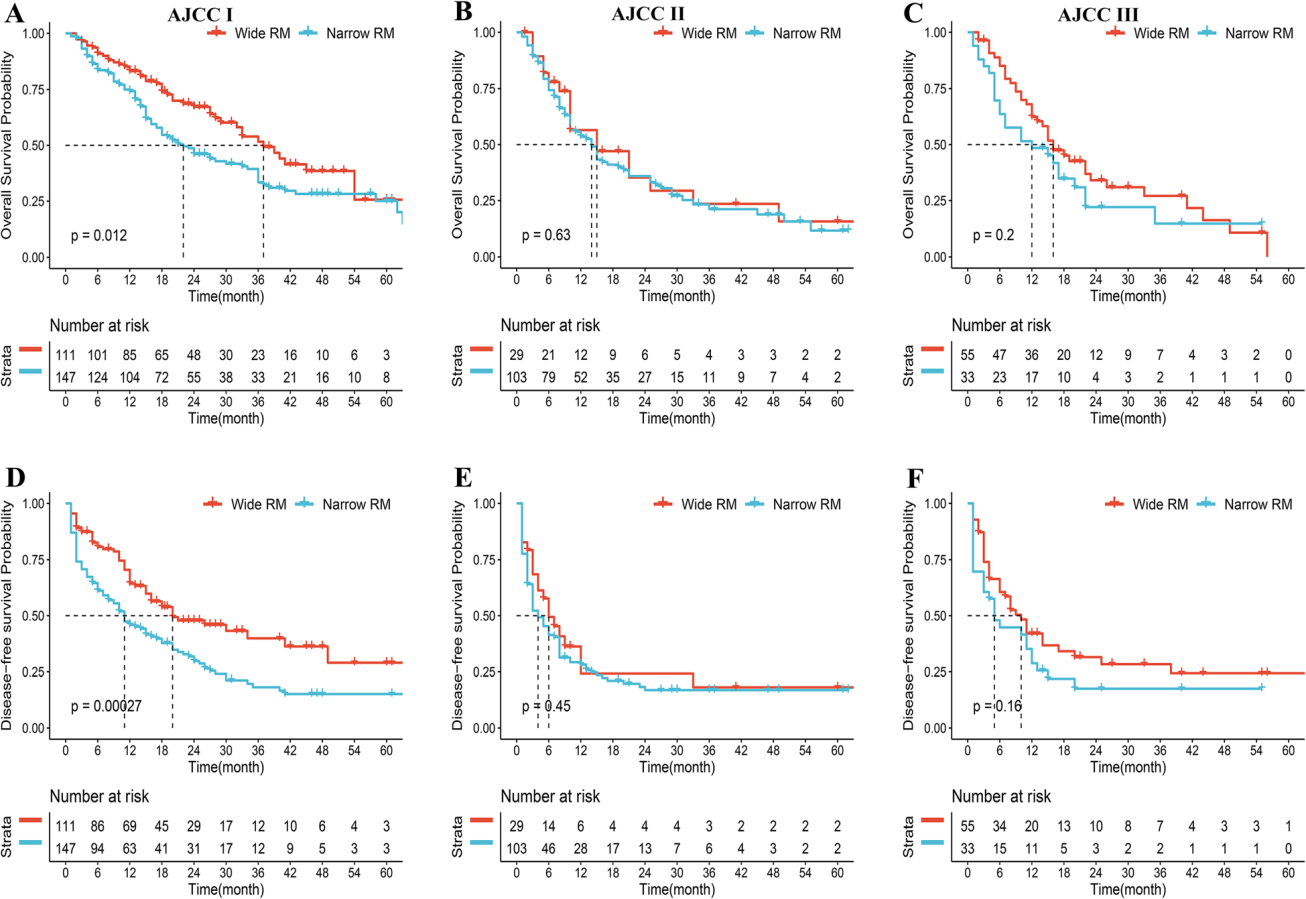
Subgroup analyses of overall survival and disease-free survival in ICC patients with AJCC8th stage I (**A**, **D**), stage II (**B**, **E**), and stage III (**C**, **F**) who underwent wide margin hepatectomy and narrow margin hepatectomy

To comprehensively understand the relationship between clinicopathological features and surgical margin, subgroup analysis was further conducted based on the 8th AJCC staging system. In total, there were 258 (54.0%), 132 (27.6%), and 88 (18.4%) patients were assigned to stage I / II / III groups. The impact of the surgical margin width depended on the context. As for stage I, patients underwent NMH had an inferior OS and DFS than patients underwent WMH (median OS was 37 vs 22 months, *P*<0.05, Fig. 5 A; median DFS was 20 vs 11 months, *P*<0.05, Fig. 5 D). However, we did not observe a significant difference between the WMH and NMH in terms of OS and DFS for ICC patients with stage II or III (Stage II: median OS was 15 vs 14 months, *P* = 0.63, Fig. 5 B; median DFS was 6 vs 4 months, *P* = 0.45, Fig. 5 E; Stage III: median OS was 16 vs 12 months, *P* = 0.20, Fig. 5 C; median DFS was 10 vs 5 months, *P* = 0.16, Fig. 5 F).

## Discussion

For ICC, liver resection remains the most effective treatment strategy at present. While surgical margin status was identified as a prognostic factor, the impact of surgical margin width on long-term outcome following R0 resection of ICC has been less well studied and remains controversial. In addition, the biological characteristics of high heterogeneity determined the ICC patients with different clinicopathological characteristics have significantly different prognostic outcome [18, 19]. Therefore, it is necessary to further explore and discuss the prognostic value of WMH in ICC patients with different characteristics and stages. In this study, we conducted a PSM analysis using multicenter ICC data, and discovered that patients underwent WMH had better prognosis outcomes compared with patients undergoing NMH; however, subgroup analysis found that WMH improved OS and DFS in AJCC I patients, but did not improve long-term prognosis in AJCC II-III patients. To our knowledge, this is the first study to evaluate the impact of surgical margin width on the outcome of ICC with different characteristics and stages.

Previous studies have offered varied views on whether margin width improves the long-term prognosis of intrahepatic cholangiocarcinoma. Ribero M et al. [20] found that margin width had no effect on OS (*P* = 0.61) and DFS (*P* > 0.05) in patients with negative margins. In addition, Watanabe et al. [15] showed that wide margins did not improve the long-term prognosis of all patients undergoing R0 hepatectomy. However, Spolverato et al. [12] showed that margin width was positively correlated with prognosis, and the prognosis was better when margin width > 1 cm. Besides, Farges et al. [14] suggested that margin width < 5 mm was an independent risk factor for poor prognosis. A meta-analysis showed a consistent result that WMH could benefit long-term survival in patients with ICC [21].

Although it is still controversial whether WMH improves prognosis, several studies have shown that margin distance is positively correlated with improved prognosis, and the wider the surgical margin, the greater the prognostic improvement [12, 14, 22]. In this study, it was further found that this improvement is undermined by an increase in the tumor stage when the surgical margin ≥1 cm, and ICC patients with stage II-III did not benefit from WMH. We believe that this is mainly due to the highly invasive characteristics of ICC. Compared with hepatocellular carcinoma, ICC has more aggressive biological characteristics, such as bile duct invasion, nerve invasion and lymph node metastasis, which are hard to be eliminated by hepatectomy alone. At present, AJCC TNM staging is the most commonly used prognostic system for ICC, and higher AJCC staging is associated with more aggressive invasion [23].

In patients with stage II of AJCC, multiple tumors usually reflect intrahepatic metastasis, and a study of European Network for the Study of Cholangiocarcinoma (ENS-CCA) has shown that the prognosis of these patients is as poor as that of patients with extrahepatic metastasis [24]. Besides, ICC with vascular invasion is also classified as stage II, which is also reported to be the independent risk factor of prognosis for patients with ICC [18, 25]. ICC with stage III represents an extensive range of invasion and metastasis, including extrahepatic invasion and lymph node metastasis. Patients with stage II-III are likely to benefit from a R0 resection,[26] however, our result showed that a wider surgical margin could hard further improve prognosis of these patients. Several previous studies conducted subgroup analysis to evaluated the impact of WMH on the outcome of ICC. Studies of Farges et al. and Watanabe et al. documented that WMH could not provide benefit for patients with lymph node metastasis [14, 15]. Similarly, we found patients with lymph node metastasis had no benefit from WMH. This reflects that lymph node metastasis was a factor that played a fatal role for the outcome of patients with ICC and WMH is not enough to improve the prognosis of these patients. In addition to lymph node metastasis, many other factors may affect the prognosis of hepatectomy. In this study, we found WMH had a longer OS and DFS than NMH in patients with CA199 ≤ 200 U/mL, CEA ≤ 5μg/L, MF type, mild tumor differentiation, no MVI, and no perineural invasion.

In clinical practice, the operation of WMH in ICC patients would be affected by many factors, including inadequate residual liver volume, tumors adherent to major vessels. Some researchers suggested preoperative portal vein embolization could improve the resectability and increase the percent of WMH in these patients, although the following surgical delay may cause tumor progression [14]. Besides, approaches such as extended resection and vascular reconstruction were considered to improve outcome further [22, 27]. Of note, aggressive approaches used to achieve a WMH may lead to an increase in adverse events, such as liver failure and massive bleeding [15]. In this study, a higher rate of intraoperative blood loss, transfusion, and postoperative complication were observed in WMH group. Given that, we suggested that wide surgical margin is recommended to improve the long-term outcome for ICC patients with AJCC stage I on the basis of adequate preoperative preparation and ensuring surgical safety. As for patients with stage II or III, WMH alone is not sufficient to improve the survival, and adjuvant therapy and other effective treatments may still needed.

There are several limitations that should be acknowledged when interpreting this study. First, this was a retrospective study and selection bias may have been present. To mitigate this bias, we conducted PSM to match the prognostic factors between the two groups. Second, detailed surgical margin width was lacked in this database, and further subgroup analyses focused on the influence of different width groups were affected. Third, due to the data of surgical margin width was from pathological exam, the measurement of it may affected by shrinkage of pathological specimens during the production process. Therefore, it is necessary to carry out prospective multicentre studies on the basis of standard intraoperative measurement of margin width.

## Conclusions

In conclusion, we suggest surgeons should strive to achieve a wide surgical margin for ICC patients with AJCC stage I to optimize the long-term outcome. As for ICC patients with AJCC stage II or III, WMH alone could not improve the survival and more effective treatments are still needed.

## Supplementary Information

**Additional file 1: Table S1.** Univariate and multivariate analysis of overall survival and disease-free survival for patients with intrahepatic cholangiocarcinoma before PSM.

## Data Availability

All data included in this study are available upon request by contact with the corresponding author.
