# Supplementary material for: Impact of surgical margin width on long-term outcomes for intrahepatic cholangiocarcinoma: a multicenter study
Source: BMC Cancer. 2021 Jul 20;21:840. doi: 10.1186/s12885-021-08560-7 (PMC8293518; doi:10.1186/s12885-021-08560-7)
Supplement: Supplementary file 1 — Additional file 1: Table S1. Univariate and multivariate analysis of overall survival and disease-free survival for patients with intrahepatic cholangiocarcinoma before PSM. [file 12885_2021_8560_MOESM1_ESM.docx]

Table S1. Univariate and multivariate analysis of overall survival and disease-free survival for patients with intrahepatic cholangiocarcinoma before PSM

| Characteristic | Variables | OS | | | | DFS | | | |  |
| --- | --- | --- | --- | --- | --- | --- | --- | --- | --- | --- |
|  |  | Univariate analysis |  | Multivariate analysis |  | Univariate analysis |  | Multivariate analysis |  |  |
|  |  | HR (95%*CI*) | *P* value | HR (95%*CI*) | *P* value | HR (95%*CI*) | *P* value | HR (95%*CI*) | *P* value |  |
| Gender | Female *vs* Male | 0.79 (0.62-1.00) | 0.055 | 0.67(0.52-0.86) | 0.001 | 0.77(0.61-0.97) | 0.023 | 0.66(0.50-0.89) | 0.006 |  |
| Age (y) | ≤60 *vs* >60 | 0.99 (0.78-1.25) | 0.941 |  |  | 0.85(0.68-1.06) | 0.145 |  |  |  |
| HBsAg | Negative *vs* Positive 1.02（0.79-1.30） | | 0.900 |  |  | 1.16(0.92-1.46) | 0.222 |  |  |  |
| ECOG score | ＜2 *vs* ≥2 | 0.99 (0.78-1.25) | 0.942 |  |  | 0.86(0.69-1.07) | 0.183 |  |  |  |
| CA19-9 (U/mL) | ≤200 *vs* >200 | 1.17（0.87-1.57） | 0.303 |  |  | 0.81(0.60-1.09) | 0.156 |  |  |  |
| CEA (µg/L) | ≤5 *vs* >5 | 1.14 (0.88-1.49) | 0.325 |  |  | 0.84(0.65-1.09) | 0.186 |  |  |  |
| Blood loss (ml) | ≤400 *vs* >400 | 0.97 (0.73-1.29) | 0.834 |  |  | 0.80(0.61-1.05) | 0.109 |  |  |  |
| [Transfusion](javascript:;) | No *vs* Yes | 0.89（0.65-1.22） | 0.480 |  |  | 0.66(0.48-0.90) | 0.009 | 0.65(0.42-0.99) | 0.046 |  |
| Laparoscopic approach | No *vs* Yes | 0.86(0.51-1.45) | 0.575 |  |  | 0.47(0.27-0.82) | 0.008 |  |  |  |
| Major hepatectomy | No *vs* Yes | 1.47 (1.13-1.91) | 0.004 |  |  | 1.43(1.13-1.83) | 0.003 |  |  |  |
| Complications | No *vs* Yes | 1.14(0.86-1.50) | 0.355 |  |  | 0.96(0.74-1.25) | 0.783 |  |  |  |
| Resection margin (cm) | ≤1 *vs* >1 | 1.39 (1.09-1.77) | 0.007 | 1.34(1.03-1.73) | 0.003 | 1.66(1.32-2.08) | <0.001 | 1.70(1.26-2.30) | 0.001 |  |
| Tumor size (cm) | ≤5 *vs* >5 | 1.53 (1.19-1.96) | 0.001 | 1.41(1.09-1.82) | 0.008 | 1.46(1.16-1.83) | 0.001 |  |  |  |
| Tumor number | Solitary vs Multiple | 1.54 (1.20-1.97) | 0.001 |  |  | 1.54(1.22-1.96) | 0.001 |  |  |  |
| Lymph node invasion | No *vs* Yes | 1.53(1.15-2.02) | 0.003 | 1.79(1.31-2.45) | 0.001 | 1.14(0.86-1.50) | 0.369 |  |  |  |
| Mass-forming | No *vs* Yes | 0.91(0.72-1.16) | 0.443 |  |  | 0.83(0.66-1.04) | 0.102 |  |  |  |
| Tumor differentiation | Well &Moderate vs Poor | 1.00(0.75-1.33) | 0.976 |  |  | 0.75(0.57-1.00) | 0.049 | 0.59（0.40-0.89） | 0.011 |  |
| Satellite | No *vs* Yes | 1.68(1.31-2.15) | <0.001 | 1.42(1.10-1.83) | 0.007 | 1.87(1.48-2.36) | <0.001 | 1.76(1.31-2.36) | ＜0.001 |  |
| MVI | No *vs* Yes | 2.10(1.53-2.88) | <0.001 | 1.76(1.26-2.45) | 0.001 | 2.23(1.66-2.99) | <0.001 | 1.64(1.06-2.54) | 0.027 |  |
| Perineural invasion | No *vs* Yes | 0.91 (0.63-1.33) | 0.641 |  |  | 0.75(0.52-1.09) | 0.132 |  |  |  |
| p-AT | No *vs* Yes | 0.60 (0.43-0.83) | 0.002 | 0.62(0.44-0.87) | 0.005 | 0.75(0.56-0.99) | 0.043 |  |  |  |

**Abbreviations:** PSM, propensity score matching; CEA, carcinoembryonic antigen; CA19-9, carbohydrate antigen 19-9; HBsAg, hepatitis B surface antigen; MVI, microvascular Invasion; p-AT, postoperative adjuvant therapy; OS, overall survival; DFS, disease-free survival; HR, hazard ratio.
